# Characteristics of Respiratory Microbiome in COPD—A Literature Review

**DOI:** 10.3390/arm94030037

**Published:** 2026-06-08

**Authors:** Iga Ciesielska-Markowska, Katarzyna Mycroft-Rzeszotarska, Piotr Korczyński, Kaja Pulik, Katarzyna Górska

**Affiliations:** 1 Department of Internal Medicine, Pulmonary Diseases & Allergy, Medical University of Warsaw, 02-091 Warsaw, Poland; katarzyna.mycroft@gmail.com (K.M.-R.); kaja.jaskiewicz@gmail.com (K.P.); 2 Department of Pulmonology, Internal Medicine, Thoracic Oncology and Transplantology, National Medical Institute of the Ministry of the Interior and Administration, 02-507 Warsaw, Poland; drkorczynski@gmail.com (P.K.); drkpgorska@gmail.com (K.G.)

**Keywords:** bacteriome, COPD, exacerbation, microbiome, smoking, sputum induction, stable, treatment

## Abstract

**Highlights:**

**What are the main findings?**

- Each region of the respiratory tract has a distinct microbial community, with partial overlap across sites.
- Patients suffering from COPD display differences in the makeup of the airway microbiome when compared to healthy individuals.

**What are the implications of the main findings?**

- Reduced microbial diversity has been associated with lower FEV1 and may relate to COPD progression and exacerbation risk.
- Low microbial biomass, contamination susceptibility, technical variability, and methodological limitations remain major challenges in accurately characterising the lung microbiome.
- Future respiratory microbiome research is expected to drive precision pulmonary medicine through multi-omics integration, AI-based analytics, and personalised microbiome-targeted therapies.

**Abstract:**

Chronic obstructive pulmonary disease (COPD) is a respiratory disease that progressively impairs airway function. Its aetiology and clinical presentation are very complex, resulting in an unpredictable course of the disease. The most important causes include smoking and environmental pollutants. However, upper airway microbiome dysbiosis has been linked with COPD severity. Through this review, we aim to compare the microbiome of the respiratory tract between its sites, and to see if there are any significant differences in the composition of the microbial flora of patients with COPD when compared to healthy individuals. While preparing this review, the PubMed database was searched using keywords such as bacteriome, COPD, exacerbation, and microbiome. Analysis of the airway microbiome shows that the three most abundant phyla are Firmicutes, Proteobacteria, and Bacteroidetes. The severity of the disease and the selected therapeutic methods influence the ratio of Proteobacteria and Firmicutes. It has been observed that a decrease in microbial diversity resulted in lower values of FEV1 in patients and could be related with COPD’s progress and exacerbation events. While exacerbation cases need quick treatment, COPD’s complex background makes it difficult to find a singular, microbial cause.

## 1. Introduction

Chronic obstructive pulmonary disease (COPD) is a respiratory disease characterised by chronic airway inflammation, causing progressive impairment of the airway function and resulting in airflow limitation [1]. Airway obstruction leads to chronic cough, phlegm production, air trapping, and shortness of breath [1]. The disease is common, affecting approximately 10.6% of the world population [2]. The most common risk factors for COPD development include smoking tobacco products, exposure to environmental pollutants, and previous respiratory infections [1]. These factors also contribute to one of the most dangerous events that patients with COPD suffer through—the exacerbation of the disease. Exacerbations can occur during any stage of airflow limitation and, if not properly handled, can lead to premature death [3].

The disease has complex clinical presentations and courses. Based on clinical presentation, symptoms of this disease, such as chronic cough with phlegm production, occurrence of exacerbations, and presence of emphysema, can be divided into specific phenotypes [4]. Unsurprisingly—just like many pathways in the human organism—factors associated with COPD development and exacerbations may interact with each other, including through changes in the airway microbiome [5]. As it has been documented already, the bacteria inhabiting the respiratory tract differ between the patients, but different stages of the disease can be characterised with changes in the composition of the airway biofilm [6]. These changes can also be propagated by other factors causing COPD, such as smoking, or even by a patient’s individual immune response [5,6]. The aim of this narrative review was to characterise the microbiome composition at different respiratory levels and compare the most common methods of obtaining microbial samples from the airway.

## 2. Microbiome Sampling

The airways form a complex system of different sites of the respiratory tract that harbour spatially distinct microbial communities [7,8,9]. Each region of the respiratory tract can be sampled by different diagnostic means. However, each sampling method is associated with disadvantages and possible side effects. Most methods require specialist materials, properly trained personnel, and are available only in an in-patient setting [5,10]. The non-invasive methods of sample collection, which can be obtained with minimal risk to the patient’s wellbeing, include oral and nasal swabbing, oral washings, and induced or spontaneous sputum collection. The invasive methods include bronchoscopy (during which one can collect BAL, cytology, and protected specimen brushings) or surgery. Below, we present the available methods of sample collection for microbial analysis.

### 2.1. Non-Invasive Sample Collection Methods

#### 2.1.1. Oropharyngeal Swab Sampling

Cotton swabs are usually used to sample the posterior wall of the oropharynx. Sometimes swabbing is also done from other areas in the oral cavity. The oral microbiome varies significantly between different microhabitats and swabs must be taken from the same site to be reliable [11]. To sample oral washings, water or mouthwashes are used; patients are asked to rinse their mouth for 30 s and then expectorate into a sample tube [12]. Saliva samples are obtained by asking patients to accumulate saliva in the mouth and spit it into a sample tube over the course of one to several minutes [12,13]. Nasal swabs are inserted into the nostrils to a depth of 1–1.5 cm and rotated on the nasal lining [14]. When the microbial analysis is postponed, samples should be subsequently frozen at −80 °C, but the time between collection and freezing varies from almost immediately to two hours between the studies [11,12,13,15].

Relative abundances of taxa have been shown to vary significantly between different sampling methods in studies performed on healthy individuals [15]. Oral swabs have lower alpha diversity than saliva samples and oral washings, while the latter two are comparable in terms of alpha and beta diversity [12,13,15]. The nasal microbiome demonstrates lower alpha diversity than the oral microbiome [16]. Oral microbiome shows higher diversity when compared to sputum samples, but is generally comparable in terms of taxa detected [15,17].

#### 2.1.2. Induced Sputum

Induced sputum samples (ISS) are obtained by using nebulised hypertonic saline (NaCl). The procedure of sputum induction consists of hypertonic saline inhalation at room temperature with increasing concentrations (3%, 4%, and 5% solutions) via an ultrasonic nebuliser. Then, the patient is encouraged to cough deeply and expectorate. Obtained sputum samples should be collected in a sterile tube for later analysis. As the procedure of sputum induction is performed mostly in patients with obstructive disorders, it should be preceded by inhalation of 400 μg of salbutamol to prevent further bronchospasm caused by inhalation of saline [18]. It should be noted that sputum induction is an established standard for studying many respiratory diseases, including COPD patients during stable states, as the specimen quality is good in terms of cell viability and reproducibility [19,20]. The main disadvantage of sputum induction is poor availability of specialist utensils, such as ultrasonic nebulisers, and trained staff outside the research or academic centres, thus making it difficult to access by patients in primary care.

#### 2.1.3. Spontaneous Sputum

Spontaneous sputum is collected during a productive cough. It is easily accessible and does not require the access to specialistic equipment or trained staff. Spontaneous sputum samples (SSS) have been used in several studies since they are easier to obtain, especially during acute exacerbations of COPD (AECOPD) when the intensity of sputum production increases, and may be preferred by some, as sputum induction might worsen airway obstruction [21]. However, SSS cannot be collected in patients unable to spontaneously expectorate sputum.

#### 2.1.4. Comparison Between ISS and SSS

Although both spontaneous and induced sputum undergo a similar path during expectoration, there are some possible explanations for dissimilarities in their microbial composition. It is a known fact that different species cultivate in different parts of the respiratory tract [7,22], possibly due to distinction of ventilation–circulation ratios in the upper and lower parts of the respiratory tract or its structure. SSS may be better at reflecting proximal airways while ISS may resemble distal parts. Therefore, SSS and ISS should not be used interchangeably [8]. Table 1 presents the key differences between ISS and SSS.

### 2.2. Invasive Sample Collection Methods

Bronchoscopy is an endoscopic procedure performed transnasally or transorally in both diagnostic and therapeutic purposes [23]. It requires the use of topical anaesthesia, preferably lidocaine, to reduce the occurrence of cough reflex or stridor due to bronchoscope insertion [24]. Despite its invasive character, it is considered an emerging gold standard for pulmonary microbiome sampling [25]. Samples for microbiome analysis can be obtained during bronchoalveolar lavage (BAL) and cytology or protected specimen brushing. It should be noted that some COPD patients might have a higher risk of complications resulting from bronchoscopy, such as desaturation, bronchoconstriction, or even pneumonia [24]; thus, COPD treatment should be optimised prior to the examination.

#### 2.2.1. Bronchoalveolar Lavage

Bronchoalveolar lavage (BAL) is a commonly used method of sampling the lower airways [26]. Although BAL collection is fairly invasive, its major advantage is that it is less prone to contamination compared to induced sputum [27]. Although the lung is colonised by the oropharyngeal microbiome through microaspiration and mucosal dispersion [28,29], the diversity of lung microbiome in BAL was found to be lower than in the upper respiratory tract (URT) [30]. Moreover, it has been found that BAL is enriched in URT and oral microbes to a greater extent in COPD patients compared to controls [29]. The enrichment of URT and oral microbes is probably caused by the impaired mucociliary clearance system in the bronchi of COPD patients [31].

In one study, the alpha diversity decreased with decreasing sample exposure to potential oral and bronchoscope contamination [32], though this was contradicted by another study where BAL samples showed higher alpha diversity compared to sputum samples. However, this study had a small sample size of only 6 patients, which may have affected the results [33].

#### 2.2.2. Cytology Brushing

Cytology brushing samples for microbiome analysis are typically collected from the sixth- to eighth-generation airways using a cytology brush [34].

#### 2.2.3. Protected Specimen Brushing

Protected specimen brushing (PSB) is another method of obtaining samples representing microbiome from the lower respiratory tract [35]. This method requires using double-sheathed, wax-plug-protected specimen brushes, which are inserted through a bronchoscope [36]. It is necessary to remember that this sample collection method is still burdened by possible contamination with foreign bacteria. However, the contamination limits itself to the outside area of the bronchoscope because the apparatus must pass through the mouth and pharynx. If the sampling procedure is performed with a mouthguard and without suction, then the contamination risk is minimal [25,35].

#### 2.2.4. Lung Explant Tissue Excision

Tissue excision, while invasive, remains an important method to consider when evaluating approaches to airway sample collection. This method is fraught with risks of complications and damage to patients’ overall health—for instance, pneumothorax or bleeding [25]. Moreover, this procedure involves specialised equipment and trained staff. Interestingly, depending on the samples’ localisation, the presence of different bacterial communities was observed, which leads to the assumption that local microarchitecture of airways has an impact on microbiome composition [7,37,38].

### 2.3. Microbiome Methodology Limitations

The lung microbiome is characteristically low in biomass, often containing very few bacterial cells, which renders it highly susceptible to contamination and technical artefacts [39]. In such samples, even trace amounts of contaminant DNA from reagents (e.g., extraction kits, polymerases, and sequencing reagents) can dominate the signal, frequently introducing spurious taxa, such as *Ralstonia*, *Pseudomonas*, or *Bradyrhizobium* [40]. Negative controls (reagent-only and sampling blanks) are essential yet inconsistently employed or reported, leading to inflated diversity or spurious taxa [41]. Batch effects arising from different DNA extraction protocols (bead-beating intensity, lysis buffers, column vs. magnetic bead purification) introduce substantial technical variability in lysis efficiency for Gram-positive vs. Gram-negative bacteria and fungi, confounding biological signals [42].

In addition, 16S rRNA gene amplicon sequencing remains a commonly used and cost-effective approach for profiling pulmonary microbial communities. However, 16S offers limited taxonomic resolution (often genus level at best, rarely species or strain), cannot distinguish viable from non-viable organisms (amplifying extracellular or dead DNA), and suffers from primer bias and copy-number variation. Shotgun metagenomics provide higher resolution, functional potential, and better viability proxies when combined with PMA (propidium monoazide) or RNA-seq, but it is more expensive, requires deeper sequencing for low-biomass samples, and remains sensitive to host DNA contamination [39]. Sequencing depth directly influences observed diversity; insufficient depth under-samples rare taxa, while over-sequencing amplifies noise in contaminated samples. Finally, microbiome data are compositional (relative abundances summing to 1), introducing spurious correlations and making absolute quantification or cross-sample comparisons difficult without spike-ins or absolute abundance methods. These issues collectively demand rigorous bioinformatics (e.g., decontamination tools like decontam or SCRuB), standardised protocols, and cautious interpretation, especially when linking pulmonary dysbiosis to chronic diseases [39,41].

## 3. Airway Microbiome

### 3.1. Microbiome of the Oral Cavity and Upper Airways

The oral cavity hosts hundreds of species of bacteria, as well as microeukaryotes, archaea, and viruses, making it the second area in the human body with the richest and most diverse microbiome, preceded only by the gut [43]. The oral cavity contains several niches, such as teeth, mucosa, tongue, and hard palates, each enriched by different bacteria [44,45].

The upper respiratory tract comprises the nasal cavity, nasopharynx, and the part of the larynx located above the vocal cord. The nasal cavity and its lining epithelium are the first-line barrier against pollutants, microbes, and allergens, which are inhaled during inspiration [46].

The bacterial composition of the oral and nasal cavities is subject to external factors. While the oral microbiota is mainly influenced by lifestyle habits, including diet and cigarette smoking [47], the nasal microbiota is mainly exposed to environmental factors, such as pollutants and airborne microorganisms [46]. The nasal microbiome is distinct from the oral microbiome. The nasal cavity bacterial communities are dominated by phyla *Actinobacteria*, *Firmicutes*, and *Proteobacteria* [48].

Since oral bacterial populations act as sources for the lung microbiome, oral dysbiosis has been linked to diseases of the lower respiratory tract, including COPD [44]. The upper respiratory tract microbiome of smokers with COPD differs from that of smokers without COPD. It was found that the abundance of *Veillonella* in the throat was increased in patients with COPD, whereas the abundance of *Streptococcus* was decreased compared to smokers without chronic obstructive pulmonary disease [49]. The presence of specific bacteria can be associated with better or worse lung function in COPD. *Veillonella* abundance in the throat was found to be negatively correlated with FEV_1_%pred, but the abundance of *Streptococcus* was found to be positively correlated with FEV_1_%pred [49]. In a different study, the abundance of *Clostridiales* was higher among those with low FEV_1_ (<lower limit of normal (LLN)) when compared to normal FEV_1_ (≥LLN), whereas abundances of several genera (*Achromobacter*, *Moraxella*, *Flavitalea*, and *Helicobacter*) were lower [50].

As mentioned before, smokers have higher abundances of periodontal bacteria [51] and therefore have a higher periodontitis risk [52]. There is an association between COPD and periodontitis [53,54]. Periodontitis is a polymicrobial disorder affecting the tissues supporting the teeth [55]. In patients with periodontitis and COPD, the genera more frequently detected in saliva compared to patients with periodontitis alone or controls were *Rothia*, *Actinomyces*, *Fusobacterium*, and *Veillonella* [56]. It was found that subgingival microbial diversity associated with periodontitis may be a risk indicator for reduced respiratory function [57]. In a murine study, it was found that oral periodontal pathogens (*Fusobacterium nucleatum*) could induce COPD-like lung changes, such as an increased number of inflammatory cells and bullae formation in the lung tissue sections, as well as worsening of the lung function indices [58].

### 3.2. Microbiome of the Lower Respiratory Tract

Moving farther inward into the airways allows us to create a profile of lung microbiome. As we pointed out above, the airways form a very complex system harbouring spatially distinct microbial communities [7,8,9], and only by choosing a correct sampling method can we truly assess the microbiome of a selected site. Clinically, this means that the way in which a sample is collected might give information about the microbiome of a specific site of the airways. When examining patients with stable COPD, it can be noted that the differentially abundant bacterial phyla in BAL are *Proteobacteria*, *Firmicutes*, *Bacteroidetes*, *Actinobacteria*, and *Fusobacteria* [29,59,60]. The dominant genera include *Streptococcus* [29,32,59], *Veillonella*, *Prevotella*, *Gemella* [31], *Pseudomonas*, *Ralstonia*, *Escherichia*, and *Rothia* [29]. Moreover, patients with COPD have increased abundance of *Haemophilus* compared to controls [32]. Studies regarding relative abundance of other genera are less consistent. *Campylobacter* [61], *Streptococcus*, and *Lactobacillales* [62] were found to have increased abundance in COPD patients compared to controls. Those results are partially contradicted by another study, which found Streptococcus equally abundant in COPD in comparison to healthy individuals [32]. In the SPIROMICS study, however, it was found that overall lung bacterial composition was not associated with COPD diagnosis but rather with lung function measures and symptom burden. Only two bacterial taxa were significantly positively associated with COPD status (*Streptococcus* and *Lactobacillales*) [62].

In the MicroCOPD study [25,63], researchers assessed results from protected specimen brushing samples taken from left upper lobe (LUL) and right lower lobe (RLL). By collecting protected sterile brushings (PSB), the authors were able to confirm that the airways were colonised by *Firmicutes*, *Actinobacteria*, *Bacteriodetes*, and *Proteobacteria* and that the most prevalent were *Streptococci*, *Veilonella*, and *Haemophilus* [63].

Studies are not consistent on alpha diversity of lung microbiome in COPD patients, with some reporting decreased alpha diversity when compared to healthy individuals [25,32] while others found no significant differences [29,64]. Notably, lower alpha diversity is a marker of microbial dysbiosis and is associated with increased airway inflammation and higher mortality in COPD [34]. No differences in alpha diversity were noted depending on current smoking status [32,34,62].

**Figure 1:**
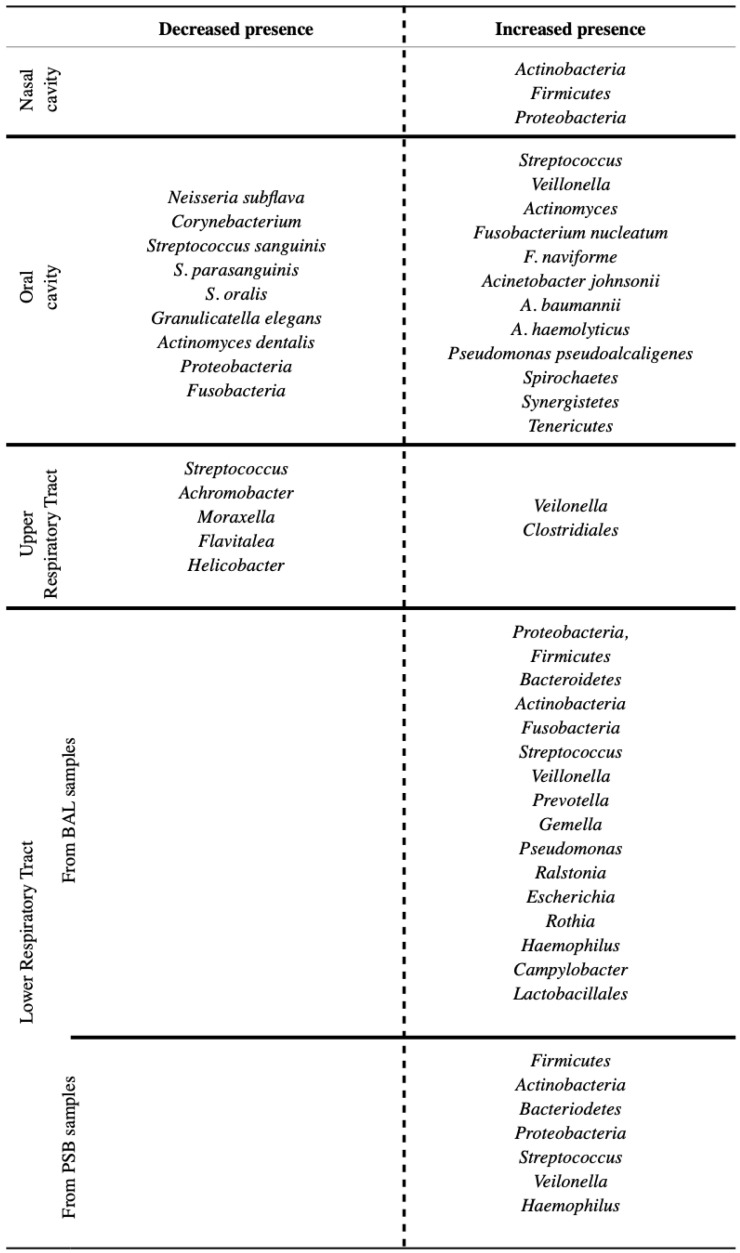
Observable changes in the abundances of microbes based on the respiratory tract location.

A thorough summary of the observed differences in microbiome composition in relation to airway levels are shown in Figure 1, located below this section. It illustrates the most commonly reported bacterial taxa across different regions of the respiratory tract.

## 4. Microbiome Composition Differences

Since it has been noted that oral bacteria communities are present in the lower respiratory tract samples, further research is necessary to establish a standardised method of collecting airway samples with minimal contamination from the oral cavity [9]. These differences in region-specific microbiomes pose a clinically interesting and important challenge. There are noticeable trends in the composition changes based on the location of the airway sampled—when comparing proportions of bacterial phyla, the oropharynx shows a difference in the amount of *Ralstonia*, *Bosea*, and *Haemophilus* than in the lung microbiome [65]. As it has been previously shown, areas of skin affected by dermatitis differ in microbial communities from healthy areas, and that the teeth in patients with periodontal disease have different microbiomes than in the disease-free patients [7]. These dependencies lead to the conclusion that there could potentially exist similar interactions in the lungs that we are yet to discover. 

Analysis of the airway microbiome shows that the three most abundant phyla are *Firmicutes*, *Proteobacteria*, and *Bacteroidetes*, and the ratios of each differ slightly between papers [9,66,67,68]. However, authors note that there were differences in the proportions of these bacteria depending on the region of the airways and the method of sample collection used; for example, *Firmicutes* were the most prevalent in bronchial wash samples [32], while *Bacteroidetes* were the most common in lung and throat swabs [49,69]. When assessing the microbiome present in sputum samples taken from COPD patients, it becomes apparent that, among the different bacteria present, there exists an overrepresentation of some specific taxa. According to the literature, these are mainly *Streptococcus*, *Haemophilus*, and *Veilonella* [70]. It has also been observed that, depending on the sample’s origin, a significant bacterial count gradient was observed, followed by counts in corresponding airway tissue and parenchymal tissue, which had the lowest microbial biomass [37]. The reason behind the gradient phenomenon and lowered bacterial count in parenchymal tissue is due to its advanced, emphysema-like destructive presentation in COPD patients, which creates a less hospitable microenvironment for respiratory microbiota development [7,37].

Evaluating the lung microbiome requires consideration of factors that impact both the local airway environment and the organism as a whole. Air pollution, a recognised aetiological factor in chronic obstructive pulmonary disease, has been shown to significantly alter the composition of the respiratory microbiome. Exposure to high concentrations of PM2.5 and/or PM10 pollutants, for as few as three consecutive days, leads to an increased abundance of *Staphylococcus*, *Haemophilus*, *Streptococcus*, and *Moraxella* [71]. Furthermore, multiple studies have indicated that exposure to air pollutants increases the risk of Pseudomonas aeruginosa infections [71,72]. This susceptibility may be attributed to alterations in the commensal microbiome; specifically, a reduction in the abundance of *Campylobacter*, *Capnocytophaga*, *Prevotella*, *Fusobacterium*, *Corynebacterium*, and *Veillonella* is negatively correlated with the levels of both environmental and household air pollutants [71,72]. Moreover, extra-pulmonary factors, such as diet and psychological stress, can modulate both the airway microbiome and the host’s susceptibility to respiratory infections. Elevated serum iron levels have been associated with a decrease in lung microbial richness, a higher abundance of *Bacteroides* spp., and the induction of pro-inflammatory responses [73]. Additionally, chronic psychological stress has been linked to the loss of alveolar macrophage homeostasis, thereby increasing infection risk. This phenomenon has been traced to gut dysbiosis, where altered production of microbiota-derived gamma-aminobutyric acid (GABA) impacts alveolar macrophages. GABA sensing via GABA receptors modulates cellular mitochondrial metabolism, directly influencing macrophage function [74]. It should be noted that the lung microbiome interacts with host immune responses in a complex bidirectional manner, although detailed immunological mechanisms are beyond the scope of this review.

### 4.1. Differences Between Stable and Exacerbated Cases of COPD

Inflammation and exacerbations in COPD can be associated with host–microbiome interplay, where, according to most findings, *Haemophilus* is responsible for host’s immunological response in stable states of the disease, as well as during exacerbations [21,75]. One way of directly influencing the inflammatory response in the airways is the synthesis of pro-inflammatory agents. In the case of COPD, it has been documented that members of the *Haemophilus* and *Moraxella* genera are able to produce lipopolysaccharide (LPS)—an endotoxin that builds the outer cellular wall of Gram-negative bacteria [9,70]. Higher values of LPS concentration can be observed in patients with a high risk of exacerbation. These changes, although slight, could prove important in exacerbation cases due to potential airflow limitation, enhanced pulmonary inflammation, and decreased FEV1, resulting from interactions between the host and LPS [9,21,70]. 

Moreover, it appears that a patient’s individual risk of exacerbation can be dictated by the composition of the microbial communities. It was observed, for instance, that *Streptococcus*, *Prevotella*, and *Gemella* have higher presence in the microbiome of patients with a low risk of exacerbation when compared to those with a high risk of exacerbation [9]. Another factor that might impact the microbiota are the treatments administered to each patient. It was observed that the severity of the disease and recurrent use of antibiotics may influence the microbiome observed in advanced COPD [76]. Antibiotic treatment resulted in an increase in *Streptococcus* and a decrease in *Haemophillus* and *Moraxella*. These trends, however, were reversed in patients that were instead treated with corticosteroid only [70].

No significant differences in alpha diversity were observed in stable COPD between patients with and without subsequent exacerbations [59]. On the other hand, it has been reported that patients with microbiomes dominated by *Streptococcus* and *Rothia* had a lower frequency of exacerbations. This group of patients was characterised by high bacterial biomass, elevated expression of microbial genes involved in metabolism and biosynthesis, and activation of the Th17 immune response. Conversely, patients with a preponderance of *Pseudomonas* had a higher risk of exacerbation and a lower proportion of macrophages, but a higher proportion of lymphocytes [29]. Patients with previous exacerbations had a higher prevalence but lower abundance of *Actinomyces graevenitzii* [59].

The majority of acute exacerbations of COPD are attributed to respiratory infections [77], and emerging evidence has highlighted specific microbial patterns associated with these events. Notably, comparative analyses of sputum samples from patients with frequent versus infrequent exacerbations revealed significant differences in alpha diversity. Patients classified as frequent exacerbators exhibited lower alpha diversity during periods of clinical stability, even in the absence of recent antibiotic or corticosteroid therapy [78]. Furthermore, any perturbation to the established microbial community can precipitate exacerbations. Current evidence suggests that such ecological disturbances may facilitate the outgrowth of pre-existing bacterial strains or the introduction of novel strains of endemic species, thereby contributing to the pathogenesis of acute exacerbations [79].

### 4.2. The Impact of ICS Treatment on Microbial Composition

Another important point is the impact of inhaled corticosteroids on the lung microbiome in COPD patients. According to GOLD 2026 recommendations, ICS are indicated in patients with concomitant asthma or exacerbations and increased blood eosinophil levels (GOLD 2026—[1]). However, the use of ICS (independently of the molecule) in COPD patients has been linked to an increased risk of pneumonia [68,80] and it is therefore advised to use these medications with caution. Interestingly, the data on the effect of ICS on lower respiratory tract microbiota in COPD patients are inconsistent [34,60]. In a DISARM study, it was shown that over 12 weeks of treatment, fluticasone propionate/salmeterol was associated with a significant reduction in alpha diversity of the airway microbiome compared to formoterol alone [34]. In a different study, no differences in alpha diversity were found between patients treated with ICS and those without ICS [60].

### 4.3. The Impact of Lifestyle on Microbial Composition

#### 4.3.1. Smoking

The effect of cigarette smoking on microbial composition in oral and nasal cavities was studied by several authors, and the results were inconsistent [51,81,82,83]. In a study by Yu et al., it was found that only in buccal mucosa was the microbiome different between smokers and non-smokers, whereas in the nasal cavity and the other seven oral sites, there were no differences [82]. Two studies found that cigarette smoke created an environment preferring strict or facultative anaerobes over strict aerobes [51,81]. Wu et al. found in oral wash an increased abundance of Streptococcus, *Veillonella* or *Actinomyces* genera in current smokers and a depletion of *Neisseria subflava* and *Corynebacterium* [81]. In a different study by Mason et al., current smokers were found to have higher abundances of periodontal and systemic pathogens (e.g., *Fusobacterium nucleatum*, *F. naviforme*, *Acinetobacter johnsonii*, *A. baumannii*, *A. haemolyticus*, *Pseudomonas pseudoalcaligenes*) and lower levels of commensals (e.g., *Streptococcus sanguinis*, *S. parasanguinis*, S. *oralis*, *Granulicatella elegans*, *Actinomyces dentalis*) in subgingival plaque when compared with non-smokers [54]. Another study on the bacterial composition in oral wash showed higher abundances of phyla *Spirochaetes*, *Synergistetes*, and *Tenericutes* and a depletion of phyla *Proteobacteria* and *Fusobacteria* in current cigarette smokers compared to never smokers [83]. Similarly, analysis of the airway microbiome in critically ill children exposed to environmental cigarette smoke revealed a higher relative abundance of *Moraxella* spp. and *S. aureus* [84]. Interestingly, the microbiota response to tobacco smoke appears to be age-dependent; adult smokers exhibited a reduced abundance of *Haemophilus* spp. [44], whereas the inverse relationship was observed in paediatric cohorts [84]. It is hypothesised that the oral microbiome remains partly resistant to cigarette smoke due to diversity and complexity of bacterial communities [85]. Of note, it was observed that oral bacteria abundances were mostly similar between former and never smokers, suggesting that specific bacteria depleted by smoking may be partly restored following smoking cessation [81].

Table 2 summarises the key findings regarding the differences in microbiome composition in response to factors such as cigarette smoking, ICS usage, and more. It can be studied beneath this section of the manuscript.

#### 4.3.2. Dietary Habits

Although data regarding the influence of dietary supplements on the adult airway microbiome remain limited, emerging evidence has highlighted the impact of maternal nutrition. Specifically, maternal supplementation with high-dose vitamin D and n-3 long-chain polyunsaturated fatty acids during the third trimester significantly altered the airway microbiota of one-month-old infants, characterised predominantly by a reduction in *Gemella*, *Veillonella*, and *Streptococcus* species [86].

### 4.4. Effects of Microbial Diversity on Lung Function

Additionally, it has been observed that a decrease in microbial diversity and an increase in the representation of *Proteobacteria* and *Neisseria* resulted in lower values of FEV_1_ in patients, which could be related with COPD’s progression and exacerbation events [9,70]. Another marker that could potentially point towards a specific microbiome is the nature of the inflammatory response. It has been shown that episodes of either bacterial or eosinophilic exacerbations presented distinct microbial communities [70].

## 5. Clinical Implications for COPD and Future Directions in Microbiome Research

Analysing the composition of the airway’s microbiome during each stage of COPD could greatly influence the therapeutic approach in cases of exacerbation events. The available literature suggests that, although these events are diagnosed and treated as independent occurrences in most cases, there might be an underlying bacterial agent that causes and intensifies each case. It is suggested that administering only anti-inflammatory medications might not be sufficient during exacerbations and that clinicians should address the infection driving the pathologic immune response [21]. This kind of treatment could be made easier by profiling the microbiota of each exacerbation event. It has been documented that there are certain bacteria present during each of the following exacerbations, and that uncovering them would allow the proper treatment to be decided for of each new event [66].

Although not many research papers focus solely on the lung virome in COPD patients, it has been observed that there are some interactions between the patient and microbiome that can be influenced by the viral families inhabiting the airways [3]. According to one study, the most prevalent families were *Poxviridae*, *Siphoviridae*, and *Myoviridae* [67], but this should be studied further. It can be concluded, however, that the viral co-infection could be yet another factor that influences the course of COPD due to possible genes present in the virome, which could affect the bacterial metabolic pathways [67].

Future directions in respiratory microbiome research are increasingly focused on the integration of multi-omics technologies and advanced computational approaches to provide a more comprehensive understanding of airway microbial ecosystems [87,88,89]. Consequently, increasing attention is being directed toward the combined application of metagenomics, metatranscriptomics, metabolomics, and proteomics [88,89]. These multi-omics analyses enable the simultaneous investigation of microbial composition, metabolic activity, gene expression, and host immune responses, thereby offering a more detailed characterisation of respiratory diseases [88]. Among these approaches, metabolomics has emerged as a promising strategy for identifying microbial and host-derived metabolites associated with airway inflammation, immune regulation, and disease progression [89]. Such profiling of bronchoalveolar lavage fluid, sputum, or exhaled breath condensate may facilitate the discovery of novel biomarkers for diseases, such as asthma, chronic obstructive pulmonary disease, and cystic fibrosis [88,90].

Recent studies have demonstrated that integrating microbial and host transcriptomic data can improve the diagnosis and classification of lower respiratory tract infections. Similarly, metatranscriptomics provides insight into the transcriptionally active fraction of the microbiome, allowing for the identification of microbial pathways allowing to determine COPD outcome and assign disease phenotype [90]. Future investigations into the respiratory tract microbiome are anticipated to place growing emphasis on the development of precision microbiome-based therapeutics that are customised to individual microbial and immunological characteristics [5,91]. In this context, microbiome engineering strategies, including live biotherapeutic products and targeted microbial communities, may represent promising approaches for restoring microbial homeostasis within the airways [91]. Future antimicrobial stewardship programmes may integrate microbiome profiling to optimise antibiotic selection, dosing, and treatment duration according to predicted ecological impacts on the respiratory tract microbiome [92]. Furthermore, microbiome-guided antibiotic strategies are anticipated to play an essential role in reducing antimicrobial resistance and minimising collateral disruption of commensal respiratory microbiota [92].

Another rapidly developing field is bacteriophage therapy, especially for multidrug-resistant respiratory pathogens, such as *Pseudomonas aeruginosa*, *Klebsiella pneumoniae*, and *Acinetobacter baumannii* [93,94,95]. Emerging evidence suggests that inhaled phage therapy may provide highly specific targeting of pathogenic bacteria, while also preserving beneficial microbial communities and reducing selective pressure associated with antibiotic resistance [93,94]. In addition, synergistic phage–antibiotic combinations, as well as engineered bacteriophages with the capacity to disrupt bacterial biofilms, are expected to broaden therapeutic options in chronic airway diseases [93,95].

Artificial intelligence (AI) and machine learning (ML) are also expected to become increasingly pivotal in respiratory microbiome research due to their ability to process highly complex and multidimensional datasets [87,96]. Machine learning algorithms can identify microbial patterns and predictive biomarkers that may elude conventional statistical methods [87]. Moreover, the AI-driven integration of microbiome-derived, metabolomic and clinical datasets may support the development of precision medicine strategies and personalised therapeutic interventions [87,96].

Simultaneously, the discovery of microbiome-related biomarkers (such as changes in cytokine levels) represents a major future direction in respiratory medicine [97,98]. Specific microbial genera, community diversity, and lower airway phageome may provide valuable diagnostic and prognostic biomarkers for respiratory infections and infection susceptibility [91,97,98]. The integration of microbiome biomarkers into precision medicine frameworks may ultimately facilitate early disease detection, patient stratification, and individualised therapeutic decision-making [97].

**Figure 2:**
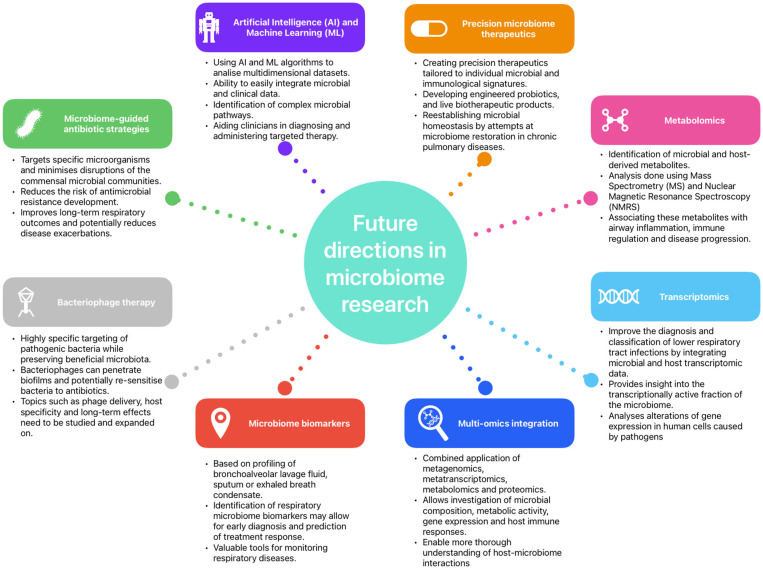
Diagram of future directions in microbiome research.

The authors of this manuscript sought to summarise the broad range of opportunities and future perspectives arising from these technological and scientific advancements, as illustrated in Figure 2 below.

## 6. Conclusions

COPD remains a health concern of worldwide significance. There is emerging evidence on the role of the microbiome in COPD pathogenesis and presentation. The lung microbiome assessment is complex and fraught with oral contamination. However, since COPD exacerbations can be driven by specific bacteria, there is a need for a quick and easy tool in microbiome assessment. There are observable cause and effect dependencies between different microorganism colonies and COPD phenotypes; however, there is a need for standardised, clinically feasible sampling, and analytical workflows to support microbiome research during exacerbations.

## Figures and Tables

**Table 1:** Comparison of key differences between ISS and SSS methods.

	Induced Sputum	Spontaneous Sputum
Represented location of the airways	Reflects distal parts more closely	Reflects proximal parts more closely
Collected cell viability	Higher	Lower
Sample contamination risk	Lower due to deep expectoration	Higher due to residual oral microbiota
Possible health risks for patients	Risk of further bronchospasm due to inhalation of saline	None
Auxiliary equipment	Ultrasonic nebuliser, hypertonic saline solutions, sterile sample cup	Sterile sample cup
Staff training requirements	Staff need to understand the procedure and follow protocols	No additional training needed

**Table 2:** Table of cited studies on lung microbiomes in COPD.

Ref.	Author	Country	Year of Study	COPD Severity in Study Population	Smoking in Study Population	Most Frequent Organisms Detected	Quantitative Changes Between COPD Patients and Healthy Individuals	Key Findings
[7]	Erb-Downward, J.R. et al.	USA	2010–2011	Moderate (30%), very severe (70%)	~45% Yes (including COPD patients and healthy smokers)	*Pseudomonas*, *Streptococcus*, *Prevotella*, *Fusobacterium*, *Veilonella*	There were no significant changes in microbial composition between study groups; decreased bacterial community diversity in patients with moderate and severe COPD compared to controls.	The diversity of the lung bacterial microbiome was lower in subjects with decreased lung function, often associated with dominance by Pseudomonas spp.; significant micro-anatomic differences in bacterial communities within the same lung of subjects with advanced COPD were noted.
[9]	Yang, C.Y. et al.	Taiwan	2015–2017	Mild-to-moderate (55.13%), severe-to-very severe (44.87%)	78.2% Yes	*Firmicutes*, *Actinobacteria*, *Proteobacteria*, *Bacteroidetes*, *Fusobacteria*	N/A	Bacterial diversity was significantly decreased in the group with a high risk of exacerbation compared to the group with a low risk of exacerbation; there were no differences in bacterial diversity or proportion of dominant bacteria at phylum and genus levels depending on airflow limitation severity.
[21]	Wang, Z. et al.	UK	2019 (Paper)	Mild (4.7%), moderate (44.2%), severe (37.2%), very severe (14%)	40.7% Yes (including COPD patients and controls)	*Veilonella*, *Prevotella*, *Haemophilus*, *Streptococcus*, *Moraxella*	Significantly increased relative abundance of Moraxella, Streptococcus, and Acinobacteria in stable COPD patients when compared to healthy subjects.	Haemophilus and Moraxella influenced different components of host immune response in COPD; while Haemophilus was associated with host responses, both in stable state and during exacerbations, the associations for Moraxella were primarily related to exacerbations.
[32]	Einarsson, G.G. et al.	Ireland	2009–2016	Mild-to-severe	N/A	*Streptococcus*, *Haemophilus*, *Rothia*, *Pseudomonas*, *Veilonella*	No significant differences in community richness were observed between COPD patients and other groups.	Bacterial community diversity was significantly lower in COPD patients than in healthy smokers and non-smokers.
[34]	Leitao Filho, F.S. et al.	Canada	2015–2021	Moderate-to-severe	46% Yes	*Firmicutes*, *Bacteroidetes*, *Proteobacteria*, *Actinobacteria*, *Fusobacteria*	N/A	Fluticasone-based ICS/LABA therapy modified the airway microbiome in COPD, leading to a relative reduction in α-diversity and a greater number of bacterial taxa changes.
[37]	Valenzi, E. et al.	USA	2018–2020	Severe-to-very severe	Yes (all participants with COPD)	*N/A*	N/A	Airway-based samples had higher bacterial loads compared to distal parenchymal tissue.
[55]	Diao, W. et al.	China	2015–2016	Moderate (53.33%), severe-to-very severe (46.67%)	Yes (all participants)	*Bacteroidetes*, *Proteobacteria*, *Firmicutes*, *Fusobacteria*, *Actinobacteria*	N/A	Veilonella was increased in COPD patients, which was negatively correlated with FEV1%pred value; Streptococcus was decreased in COPD patients, which was positively correlated with FEV1%pred value.
[63]	Leiten, E.O. et al.	Norway	2006–2007	Mild-to-moderate (62.3%), severe (37.7%)	23.77% Yes	*Firmicutes*, *Bacteroidetes*, *Proteobacteria*, *Fusobacteria*	N/A	No differences in lung microbiota composition or diversity were found that could predict future exacerbation severity in stable COPD.
[64]	Tangedal, S. et al.	Norway	2012–2015	Mild-to-moderate (69.07%), severe-to-very severe (30.93%)	30.93% Yes (including healthy controls)	*Firmicutes*, *Granulicatella*, *Streptococcus*, *Gemella*	Oribacterium was absent in smoking patients with COPD, whereas no significant difference was linked to smoking in the control group; while Alloscardovia was absent in female patients with COPD, it was found in female controls.	Authors noted a decrease in alpha diversity in COPD compared with controls; nine genera were identified to be different between patients with COPD and controls including Streptococcus; smoking quantified by pack-years was associated with a significant reduction in Haemophilus and Lachnoanaerobaculum in healthy controls.
[71]	Goolam Mahomed, T. et al.	South Africa	2017	N/A	37.5% Yes	*Streptococcus*, *Haemophilus*, *Prevotella*, *Veilonella*, *Granulicatella*	N/A	Authors did not notice any statistically significant differences in the microbiome of COPD patients, regardless of the disease state; results of this study showed differences in frequencies of certain phyla and genera (including Proteobacteria and Firmicutes) in comparison to studies from Europe and the USA.
[73]	Sze, M.A. et al.	Canada	2015	Severe-to-very severe	N/A	*Proteobacteria*, *Bacteroidetes*, *Firmicutes*, *Actinobacteria*	Density of microbiomes remain the same in patients with COPD and healthy individuals.	There was a decline in microbial diversity that was associated with emphysematous destruction, remodelling of the bronchiolar and alveolar tissue, and infiltration of the tissue by CD4+ T cells.
[74]	Wang, Z. et al.	UK	2016 (Paper)	Mild (1.15%), moderate (40.23%), severe (36.78%), very severe (21.84%)	42.53% Yes	*Firmicutes*, *Proteobacteria*, *Actinobacteria*, *Bacteroidetes*	N/A	Microbiome structure and diversity were highly correlated with sputum interleukin-8; microbial diversity is reduced and an increase in the Proteobacteria:Firmicutes ratio was observed in patients treated with steroids; a reverse trend was present in patients treated with antibiotics.

## Data Availability

No new data were created or analyzed in this study.

## Abbreviations

The following abbreviations are used in this manuscript:

COPD	Chronic obstructive pulmonary disease
BAL	Bronchoalveolar lavage
ISS	Induced sputum samples
SSS	Spontaneous sputum samples
AECOPD	Acute exacerbations of chronic obstructive pulmonary disease
URT	Upper respiratory tract
PSB	Protected specimens brushing
FEV1	Forced expiratory volume in 1 s
FEV1%PRED	Predicted value of forced expiratory volume in 1 s
LUL	Left upper lobe
RLL	Right lower lobe
LPS	Lipopolysaccharide
ICS	Inhaled corticosteroids
N/A	Not available
GABA	Gamma-aminobutyric acid
AI	Artificial intelligence
ML	Machine learning
